# Automated Electrochemical Glucose Biosensor Platform as an Efficient Tool Toward On-Line Fermentation Monitoring: Novel Application Approaches and Insights

**DOI:** 10.3389/fbioe.2020.00436

**Published:** 2020-05-21

**Authors:** Katrin Pontius, Daria Semenova, Yuliya E. Silina, Krist V. Gernaey, Helena Junicke

**Affiliations:** ^1^Department of Chemical and Biochemical Engineering, Process and Systems Engineering Center (PROSYS), Technical University of Denmark, Kongens Lyngby, Denmark; ^2^Institute of Biochemistry, Saarland University, Saarbrücken, Germany

**Keywords:** glucose biosensor, flow-through-cell, on-line monitoring, yeast fermentation, bioprocesses

## Abstract

Monitoring and control of fermentation processes remain a crucial challenge for both laboratory and industrial-scale experiments. Reliable identification and quantification of the key process parameters in on-line mode allow operation of the fermentation at optimal reactor efficiency, maximizing productivity while minimizing waste. However, state-of-the-art fermentation on-line monitoring is still limited to a number of standard measurements such as pH, temperature and dissolved oxygen, as well as off-gas analysis as an advanced possibility. Despite the availability of commercial biosensor-based platforms that have been established for continuous monitoring of glucose and various biological variables within healthcare, on-line glucose quantification in fermentation processes has not been implemented yet to a large degree. For the first time, this work presents a complete study of a commercial flow-through-cell with integrated electrochemical glucose biosensors (1^st^ generation) applied in different media, and importantly, at- and on-line during a yeast fed-batch fermentation process. Remarkably, the glucose biosensor–based platform combined with the developed methodology was able to detect glucose concentrations up to 150 mM in the complex fermentation broth, on both cell-free and cell-containing samples, when not compromised by oxygen limitations. This is four to six-fold higher than previously described in the literature presenting the application of biosensors predominately toward cell-free fermentation samples. The automated biosensor platform allowed reliable glucose quantification in a significantly less resource and time (<5 min) consuming manner compared to conventional HPLC analysis with a refractive index (RI) detector performed as reference measurement. Moreover, the presented biosensor platform demonstrated outstanding mechanical stability in direct contact with the fermentation medium and accurate glucose quantification in the presence of various electroactive species. Coupled with the developed methodology it can be readily considered as a simple, robust, accurate and inexpensive tool for real-time glucose monitoring in fermentation processes.

## Introduction

Bioprocess manufacturing has played a key role in food, pharma and the chemical industry for the last 50 years. The producing core of any biotech industry is the fermentation process itself which is often considered as the most complex unit operation within bio-manufacturing. However, bioreactors for both laboratory and industrial scale experiments are rather sparely equipped with supporting monitoring tools, generally only involving standard sensors such as pH, temperature and dissolved oxygen (Flickinger, 2010; Pohlscheidt et al., 2013). On-line process data of the critical process parameters such as substrate and product concentration is often lacking and thus, manual manipulations based on experience instead of process data are frequently used to operate the process at the desired set-point. In order to overcome current limitations and establish new standards required for modern bioprocessing, both industry and academia are focusing increasingly on providing advanced monitoring tools enabling appropriate control strategies allowing integrated process efficiency.

Glucose is a major carbon and energy source in the fermentation industry and as such, evidently, monitoring and control of glucose concentrations during fermentation processes is beneficial for any feeding strategy, optimizing biomass production itself as well as the production of metabolites such as amino acids, alcohols, peptides and proteins. Despite the importance of glucose for various fermentation processes, commonly accepted tools for glucose monitoring are not implemented yet. Generally, the measurements of relevant fermentation parameters such as glucose and other substrate and product concentrations are performed by means of spectroscopic and chromatographic techniques. These methods are considered resource and time intensive and as such not suitable for prompt analysis or continuous monitoring applications. Some fully automated systems for multicomponent analysis, including biosensor based technologies as presented in this study, were developed for rapid quantitative analysis significantly reducing measurement time and operational errors (e.g., Cedex Bio from Roche Diagnostics GmbH, the Biochemical Analyzer series from Yellow Springs Instruments (YSI, United States), the BioProfile series from Nova Biomedical, the Analyzer series from SBA (China), and BioPAT^®^ Trace and BioPAT^®^ Multi Trace sold by Sartorius). Some of these devises even enable on-line monitoring and control. The application of online glucose monitoring and glucose feed control by such a biosensor based analyzer system was shown to bring considerable advantages in bioprocess development (Moeller et al., 2011, 2010). Moeller and coworkers successfully connected the system ProcessTRACE from Trace Analytics GmbH (Braunschweig, Germany) to the control software of the bioreactor, and thus could control the glucose level at the desired set point by means of a P controller. Notably, glucose monitoring and feed control was achieved during long-term repeated fed-batch fermentations lasting nearly 600 h (Moeller et al., 2011), without any notable decrease in the biosensor activity.

However, it is worth mentioning that an auto-sampling unit facilitating cell-separation is part of the ProcessTRACE system and thus, glucose monitoring is performed quasi-online on cell-free samples. Generally, notwithstanding their great performance, such advanced analyzer systems are rather expensive investments and some of them occupy considerable laboratory space, which renders them less attractive for integration as process monitoring tools in every laboratory.

Continuous glucose monitoring is probably most advanced in the field of healthcare applications. Numerous glucose biosensors were developed and introduced on the market toward health care and clinical diagnostics, mainly for diabetes management and treatment (Wang, 2008; Chen et al., 2013; Moser and Jobst, 2013). Nowadays, sophisticated biosensor solutions are commercially available and integrated in designs suitable for the application to fermentation processes. Yet, outside China, they have been broadly overlooked for the monitoring and control of fermentation processes (Yan et al., 2014). Apart from being compact, relatively cheap, simple to handle and quick to fabricate (i.e., screen printing and thin film deposition methods, Jobst et al., 1996), biosensor technologies have been fully validated with biological samples and can provide all features required for fermentation monitoring (i.e., blood samples demonstrate comparable matrix complexity as fermentation broth). Moreover, the presence of a specific bioreceptor guarantees highly selective detection of the desired molecule in the complex fermentation matrix. Enzyme based biosensors (mainly glucose biosensors containing glucose oxidase (GOx) as a bioreceptor) were introduced more than 50 years ago by Clark and Lyons (Clark and Lyons, 1962). Subsequently, the first concept of an autoclavable and regenerable glucose biosensor for on-line monitoring of glucose in fermentation media was proposed in 1987 (Brooks et al., 1987). Since then, the potential of the biosensor concept suitable for continuous glucose monitoring in fermentation processes was demonstrated by various research groups (Rishpon et al., 1990; Kauffmann and Pravda, 1998; Mehrvar and Abdi, 2004; Chen et al., 2013; Tian et al., 2014; Hwang et al., 2018) and the lack of research devoted to studying and applying biosensors as a fermentation monitoring tool was strongly discussed in literature in the recent years (Yan et al., 2014; Bahadir and Sezgintürk, 2015; Mehrotra, 2016; Shi et al., 2017). Contrarily, although the number of glucose biosensor relevant articles published in the last 15 years has maintained an increasing trend (Hwang et al., 2018), as has the variety of novel sensor designs, their practical application toward fermentation processes has stagnated. The stagnancy observed toward fermentation application might be explained by concerns regarding long term stability of the enzyme, a narrow detection range of available biosensing technologies (mainly up to only 25 mM due to relevant blood sugar levels in diabetes patients), sterility or simply, because no satisfying, ready-to-use commercial solutions have been available applicable to bioreactors. It is important to note that clinical diagnostics require certainly different integration strategies compared to biotech applications. By now, the recent innovations in glucose biosensor technology include miniaturized biosensor arrays for simultaneous detection of up to four components (Moser and Jobst, 2013; Kamanin et al., 2015), their fabrication as a flow–through-unit (Kanso et al., 2017), as well as enzyme free glucose sensors mimicking enzyme specificity (Ampelli et al., 2015). Nevertheless, the majority of sensors described previously exhibit a linear glucose detection range limited to a concentration of 33 mM and are mainly described as a subject of scientific interest rather than an application as an on-line monitoring tool for fermentation processes.

Herein, we introduce a commercial miniaturized sensing platform for continuous monitoring of glucose applied for the first time to fermentation processes. To this end, the available biosensor acquired from Jobst Technologies GmbH – an IST AG company with integrated 1^st^ generation glucose biosensors was adapted, applied and validated for glucose quantification in yeast fermentation samples. Notably, combining advantages such as multi-array design and flow-through integration, the presented platform allows enhancing the detection range of glucose concentrations up to 150 mM. More importantly, the rapid and simplified glucose quantification by means of the presented biosensor platform was highlighted for both at-line and on-line measurements during a yeast fed-batch fermentation. Finally, besides accurate quantification of glucose in fermentation samples, the biosensing platform was approved with outstanding mechanical stability in the presence of the complex fermentation broth and can be readily considered for fast at-line and continuous on-line monitoring of glucose in fermentation samples (cell-free and cell-containing) showing excellent robustness, compactness and simplicity.

## Materials and Methods

### Working Principle and Operation of the Biosensor Platform

**FIGURE 1:**
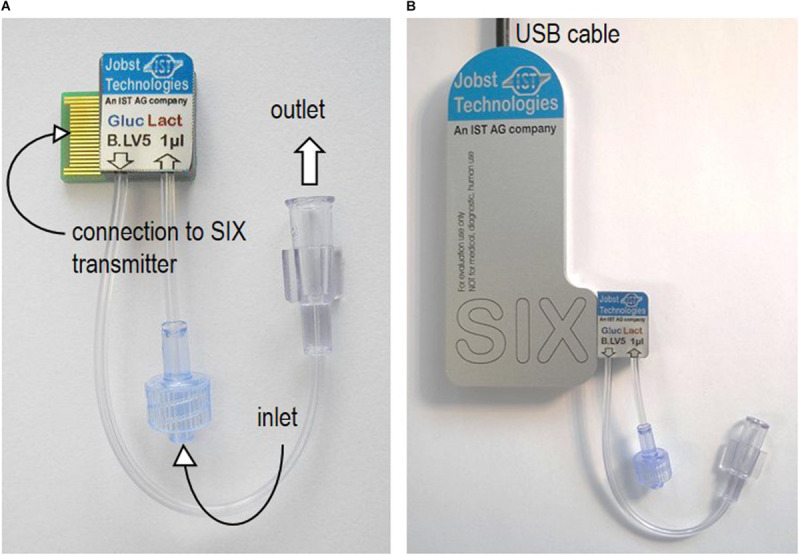
Biosensor platform. **(A)** The biosensor chip B.LV5 designed as a flow-through-cell with connection to the SIX transmitter and luer fittings as connections for inlet and outlet. **(B)** The biosensor connected to the SIX transmitter.

The biosensor platform is presented in Figure 1 and consisted of the biosensor chip B.LV5 (extended range sensor, operational pH – range 5 – 9, Figure 1A), the potentiostat with customized connection for fitting the biosensor (SIX transmitter, Figure 1B), as well as the bioMON software used for operating the biosensor platform. All components are customized and provided by Jobst Technologies GmbH – an IST AG company (Freiburg, Germany). The biosensor chip is designed as a 1 μl flow-through-cell with tubing (0.5 mm inner diameter) for inlet and outlet ending in luer fittings. Hence, it could be readily integrated in a fermentation set-up.

To facilitate a flow of the sample through the biosensor, a pump (Ismatec Reglo ICC, Wertheim, Germany) was connected via tubing (Ismatec, TYGON S3TM, E-LFL, ID 1.52 mm) and the respective luer connector before the inlet of the biosensor.

Detailed information regarding the biosensor system was presented previously (Jobst et al., 1996; Moser and Jobst, 2013). Briefly, the electrochemical cell consists of two Pt -working and one Pt -counter electrodes and an internal Ag/AgCl pseudo-reference electrode. Besides, two blank (non-enzyme coated) Pt-working electrodes are included, one for each working electrode. Note that, by the multi-array design of the sensor, each sample is automatically measured in duplicate (two working electrodes for glucose detection). The chronoamperometric measurements in the presence of different media and glucose concentrations were performed at a voltage of +450 mV vs. Ag/AgCl. Subtracting the respective blank current (obtained from the respective blank electrode) from the steady-state biosensor response (current obtained from the respective enzyme coated working electrode) resulted in the current value that was correlated with the glucose concentration present^1^. All biosensor measurements were subject to a standard deviation (st. dev.) of maximal 5%.

The biosensor as such consists of a thin enzymatic layer (GOx based) together with the enzyme-bound cofactor FAD/FADH_2_ entrapped into a hydro-gel membrane that is placed on top of the H_2_O_2_ sensitive Pt-working electrode. The operation principle of the biosensor is based on the enzymatic oxidation of glucose to gluconic acid (1), followed by the re-oxidation of the flavin groups (FAD/FADH_2_) in the presence of oxygen (co-factor regeneration) resulting in H_2_O_2_ generation (2), and by anodic oxidation of the produced H_2_O_2_ (3) on the surface of the working electrode.

(1) GOx(FAD)+Glucose→GOx(FADH)2+Gluconicacid

(2) GOx(FADH)2+O→2GOx(FAD)+HO22

(3) HO2→2O+22H++2e-

The amount of H_2_O_2_ produced, or, respectively, the amount of H_2_O_2_ oxidized on the working electrode is proportional to the glucose concentration present in the sample.

### Calibration of the Biosensor Platform in Different Media

Calibration solutions were prepared in acetate buffer (the recommended buffer system from the company), yeast extract – peptone (YP) medium (the fermentation medium) and YP medium containing 9 g/l NaCl. The biosensor response in the presence of YP medium containing additional salt was studied as the Ag/AgCl reference electrode depends on a sufficient concentration of chloride ions in solution (recommended in the specification sheet are approx. 110 mM). However, the chloride concentration estimated in the YP medium was only about 5 mM, and hence, 9 g/l (154 mM) NaCl was added. This concentration of NaCl was chosen, as saline solution (9 g/l NaCl in water) is conventionally used as an isotonic non-nutritional dilution solution when handling microbial cells and thus considered to be an appropriate concentration for the fermentation samples.

The YP medium contained per liter of DI water 10 g yeast extract (Merck, Darmstadt, Germany, NaCl < 5%) and 20 g peptone (Merck, Darmstadt Germany, NaCl < 3%).

The acetate buffer contained per liter of DI water 0.313 g potassium chloride, 5.443 g sodium acetate trihydrate, 5.669 g sodium chloride, 0.014 g sodium phosphate monobasic monohydrate, 0.114 g sodium phosphate dibasic dihydrate, 0.122 g magnesium chloride dehydrate and 1 ml Proclin 300 to avoid microbial activity. Proclin is a preservative widely used for diagnostic reagents, effectively inhibiting the growth of a broad spectrum of microbes at low working concentrations, and hence helping to keep the sensor free from contamination. All ingredients were purchased from Sigma (Saint Louis, MO, United States). Note that, when referring to “buffer” within this work, it was consistently this acetate buffer solution recommended for calibration by the company.

To be in line with the operational pH of 6 used for the fermentation process, the pH in all working solutions (acetate buffer and YP solutions used for biosensor calibration) was adjusted to 6 by addition of a few drops of 5 M H_2_SO_4_, (prepared from 96% H_2_SO_4_, BASF, Ludwigshafen, Germany). pH measurements for pH adjustments were performed with the PHM22 Lab pH Meter (Radiometer Analytical SAS, Villeurbanne Cedex, France).

For each batch of solutions, the respective background matrix (buffer, YP medium and YP medium containing salt) was maintained while the sugar concentration was altered. The calibration range was chosen between 1 and 150 mM glucose concentration (starting with 1 mM, followed by 5 mM and continued in steps of 10 mM up to 150 mM), in particular challenging the biosensor platform with respect to the upper detection limit (60 mM glucose is the recommended upper detection limit by the company). Besides, aiming at fast at-line and continuous on-line glucose quantification during the fermentation process, the upper limit referred to the initial glucose concentration present in the fermentation process. The lower limit of the calibration was chosen according to the lowest glucose concentration accurately measured by the reference method (high performance liquid chromatography, (HPLC) with refractive index (RI) detector). Glucose calibration samples were quantified under static (no flow) and dynamic operation, in case of the latter applying a constant flow rate of 0.2 ml/min. Under static conditions, the sample was pumped into the biosensor, the pump was stopped and the measurement was started with the sample standing still in the measurement cell. When the measurement was finished, indicated by a final constant current (steady-state), the sample was pumped out and the next sample was pumped into the biosensor, flushing the flow-through-cell with approximately 100 μl of new sample before stopping the pump and starting the measurement of the new sample. The procedure was equal for samples measured under flow, with the difference that the pump was kept running during operation of the biosensor platform. All samples for calibration were pumped in and out of the biosensor chip one after another, with a small volume of air in between samples (indicating the end of the old/the beginning of the new sample). In case of measuring glucose concentrations in fermentation samples, the biosensor cell was left filled with buffer in between measurements.

Note that, with respect to simple and rapid at-line measurements, static operation of the biosensor platform was a matter of curiosity since minimizing the technical effort from a pump to a syringe to load the biosensor chip with sample. However, dynamic operation is clearly favorable considering the generally enhanced mass transfer under flow conditions.

The respective calibration curves obtained in YP medium with biosensor chip 1 and 2 (two biosensor chips were investigated, see below section) were subsequently applied to determine the glucose concentration of the yeast fermentation samples, at-line (biosensor chip 1) and, respectively, on-line (biosensor chip 2). In order to guarantee the accuracy of the glucose measurements provided by the biosensing platform, the calibration solutions were validated by HPLC measurements.

### Investigations of the Biosensor Platform

Two biosensor chips of the same type (B.LV5) connected to the SIX transmitter were characterized under different conditions. The first biosensor chip was studied with respect to calibration in different media (acetate buffer, YP medium and YP medium containing additional 9 g/l NaCl), at-line analysis of fermentation samples (both cell-containing and cell-free), functionality over time (calibration before and after measurements of various fermentation samples) and finally the performance of the biosensor after a 3 month storage period. In order to account for batch-to-batch variability, a second biosensor chip was investigated regarding calibration in different media, 10 h continuous operation as an on-line glucose monitoring tool during a yeast fed-batch fermentation and recalibration after usage. The experiments conducted using biosensor chip 1 and 2 are summarized in Table 1.

**TABLE 1:** Overview of the experiments conducted with biosensor chip 1 and 2.

**Biosensor**	**Experimental investigation**
Chip 1	*Investigation of the biosensor platform* (1) Calibration in different media, applying dynamic (flow) and static (no-flow) operation; (2) At-line analysis of fermentation samples obtained from *fermentation 1* (cell-free and cell-containing); (3) Recalibration after usage in (1) and (2); (4) Storage stability after (1), (2), and (3), and a storage period of 3 months.
Chip 2	*Batch to batch variability of the biosensor platform* (1) Calibration in different media, dynamic operation; (2) Continuous on-line measurements over a 10 h yeast fed-batch fermentation conducted during *fermentation 2*; (3) Recalibration after usage in (1) and (2).

### Yeast Fermentation Processes

Yeast fermentations (*fermentation 1* and *2* in Table 1) were performed cultivating the classical laboratory yeast strain *CENPK-113 7D* in yeast extract – peptone – dextrose (YPD) medium in a working volume of 2 l. The YPD medium contained per liter of water 10 g yeast extract, 20 g peptone and 20 g glucose (dextrose) (Merck, Darmstadt, Germany). The fermentation vessel was equipped with dissolved oxygen (DO), pH and temperature probe, the whole set-up controlled by an Applikon ez controller (Applikon, Delft, Netherlands). The fermentation process was run at a stirrer speed of 800 rpm, an aeration rate of 1 vvm, a pH of 6 and a temperature of 30°C. The dissolved oxygen tension (DOT) stayed above 30% of saturation throughout the fermentation processes, indicating that no oxygen limitation occurred during the cultivations. The fermenter was inoculated with 180 ml overnight culture, pre-grown in YPD medium for 12–14 h at 30°C and 180 rpm. The increase of the biomass concentration was followed by classical optical density (OD) measurements at-line^2^ and dry weight measurements off-line^3^. Additionally the increase of the biomass concentration was followed on-line^4^ by means of a backscatter cell using the non-invasive Cell Growth Quantifier CGQBIOR from aquila biolabs GmbH (Baesweiler, Germany).

The OD was determined in duplicate (st. dev. <3%) at 600 nm with the UV-1800 spectrophotometer from Shimadzu (Duisburg, Germany).

Dry weight (DW) measurements were performed in duplicate (st. dev. <5%) by filtering 5 ml of sample via a 0.2 μm filter-paper (Cellulose Nitrate Membrane Filters, Whatman, Dassel, Germany), subsequently washing the filter three times with 5 ml of purified water applying vacuum filtration. The filter cake, consisting of the washed biomass, was dried in a microwave for 15 min at 180 W. The dry weight of the sample was obtained by subtracting the mass of an empty filter paper from the mass of the filter paper containing dry biomass.

### At-Line Operation of the Biosensor Platform During a Yeast Fed-Batch Fermentation

During *fermentation 1*, at-line glucose measurements were performed with biosensor chip 1 (Table 1, Chip 1, point 2) based on the calibration curve obtained previously in YP medium, pumping each sample through the biosensor chip with a flow rate of 0.2 ml/min. Samples were withdrawn manually every hour and glucose was quantified via the biosensor platform in both cell-free and a cell-containing samples. Cell-free samples were filtered via a 0.2 μm sterile filter (Sartorius, Göttingen, Germany) prior to the measurement. Additionally, each glucose measurement obtained from the biosensor platform was supplemented with glucose quantification by HPLC. The fermentation process was followed over a 13 h period, during which glucose was fed three times (addition of 100 ml of 500 g/l glucose solution after 7, 9, and 11 h).

### On-Line Operation of the Biosensor Platform During a Yeast Fed-Batch Fermentation

For operating a second biosensor chip (Table 1, chip 2, point 2) on-line during *fermentation 2*, the platform was connected to the fermenter via a recirculation loop. The fermentation broth was circulated from a standard sampling port inside the bioreactor to the biosensor chip and back to the fermenter (using a separate inlet). Supplementary Figures S1, S2 show the biosensor platform connected to the fermenter. The dead volume inside the tubing until the biosensor chip was approximately 1 ml. A flow rate of 1 ml/min was chosen according to the dead volume prior to the biosensor ensuring a reasonable exchange of volume inside the biosensor flow-through-cell with fresh fermentation broth (within this set-up approximately every minute). According to the manual, the maximal flow rate when operating the biosensor should not exceed 1 ml/min, which is typical for microfluidic devices. During this run, the fermenter was equipped with two sampling ports, one used for the recirculation loop, the other one to withdraw samples manually every hour for the validation of the biosensor data by HPLC measurements.

#### Oxygen Consumption Inside Cell-Containing Fermentation Samples

Since in cell-containing fermentation samples dissolved oxygen is a substrate consumed by both, the cells (respiratory activity) and the enzyme layer insider the biosensor chip (co-factor regeneration), the dissolved oxygen (DO) concentration over time was measured in distinct fermentation samples using an optical oxygen sensor similar to the set-up assembly and procedure described previously by Semenova et al. (2018). Briefly, the OXR430 retractable needle-type fiber-optic oxygen minisensor (PyroScience GmbH, Aachen, Germany) was connected to a FireStingO_2_ fiber-optic meter (PyroScience GmbH, Aachen, Germany) and controlled by the Pyro Oxygen Logger software (PyroScience GmbH, Aachen, Germany). The measurement was performed at room temperature with constant stirring speed until the DO was depleted by the cells. This measurement of oxygen consumption in cell-containing fermentation samples was conducted in order to explain the observed differences in the biosensor signal stabilization, when comparing the signal development of cell-free and cell-containing fermentation samples (see Supplementary Figures S3, S4). Respectively, this experiment furthermore explains the noted differences between glucose concentrations measured in cell-free and cell-containing samples at-line, when comparing biosensor and HPLC results.

### Reference Analysis Used for Validation of the Biosensor Platform

#### HPLC With RI Detector

To verify the glucose concentrations in the calibration solutions and fermentation samples, HPLC (Ultimate 3000 Dionex, Sunnyvale, CA, United States) was utilized. Each sample was measured in duplicate with a standard deviation of maximal 2% across all measurements. The separation was achieved on the Aminex HPX 87 H column, 300 × 7.8 mm (BIORAD, Copenhagen, Denmark) operated at 50°C and equipped with Refract Max 520 RI detector. The column was operated with 5 mM H_2_SO_4_ in purified water as mobile phase and a constant flow rate of 0.6 ml/min, injecting a sample volume of 5 μl for analysis. Prior to analysis, samples were filtered via a 0.2 μm filter and acidified (950 μl sample + 50 μl 5M H_2_SO_4_) due to the ion exchange principle used for separation. The data analysis was performed with the software Chromeleon 6.8 (Thermo Fisher Scientific, San Francisco, CA, United States).

#### Gas-Chromatography Mass Spectrometry (GC-MS)

The YPD medium at different stages during *fermentation 2* was analyzed by means of GC-MS in order to demonstrate the matrix complexity of the fermentation samples. A sample volume of 2 μl was injected by a PAL auto-sampler (CTC Analytics, Zwingen, Switzerland) into the QP5050 (Shimadzu, Kyoto, Japan) GC-MS system. Between measurements, the autosampler was washed with DI water for 3 min. The injection temperature was set at 200°C (split ratio 1:100). For the separation, a ZB-WAX-plus column (Torrance, CA, United States, 30 m × 0.25 mm; film thickness 0.25 μm) was utilized at the following temperature program: started at 50°C for 1 min, then raised to 200°C at 20 K/min and held at the final temperature 250°C for 10 min. The transfer line to the mass spectrometer and the source temperatures were 220 and 200°C, respectively. The ionization of the compounds was performed at an acceleration voltage of 70 eV. Mass spectra were recorded in TIC mode at the m/z range of 40–600. All samples were measured in triplicate.

#### Inductively Coupled Plasma Mass Spectrometry (ICP-MS)

To verify the long-term mechanical stability of the metal- based biosensor chip 1 and 2 (Pt-working electrode, Pt-counter electrode and Ag/AgCl pseudo reference electrode), ICP-MS analysis of glucose solutions in buffer and YP medium was performed. For this purpose, the samples were collected after having passed the biosensor (Table 1, chip 1 and chip 2, point 3) and analyzed on the ELEMENT XR (Thermo Fisher Scientific, Bremen, Germany) coupled with the auto-sampler SC-E2 DX (Elemental Scientific, Omaha, NE, United States). Hence, if the biosensor leaked electrode material during operation, it would be detected. For this goal, to verify the stability of the working and, respectively, pseudo reference electrode, Pt_195_ and Ag_107_ isotopes were measured at high resolution (HR) mode with the following source parameters: cool gas, 16.00 l/min; sample gas, 1.160 l/min; Faraday deflection, −215 V; plasma power, 1250 W; peristaltic pump speed, 10 rpm; torch X-Pos., 2.1 mm; torch Y-Pos., 0.9 mm; torch Z-Pos., −4.0 mm. The detector was set at 1500 V. Calibration solutions and blank samples were supplemented with 2% HCl prior to ICP-MS analysis. All samples were measured in triplicate and the final results were expressed as mean values with relative standard deviation.

### Storage Conditions of the Biosensor Flow-Through-Cell

After usage, the biosensor chip was flushed first with buffer and then with DI water for approximately 10 min at a constant flow rate of 0.5 ml/min (in order to remove sample residuals potentially trapped inside the flow-through cell), and subsequently dried with compressed air. Then, the biosensor chip was stored with closed luer connections together with a desiccant (SiO_2_) in a small bag at 4°C in the dark.

## Results

### Calibration in Different Media

**FIGURE 2:**
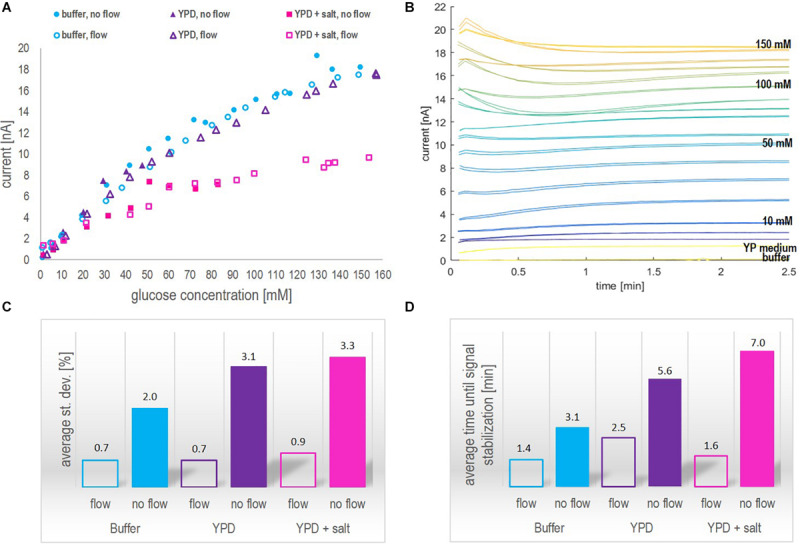
**(A)** Steady-state current in nA as a function of the glucose concentration in mM obtained in the different media applying the biosensor platform under dynamic (flow of 0.2 ml/min) and static (no-flow) operation. **(B)** Raw signal development (current in nA as a function of time in min) obtained for different glucose concentrations in YP medium under dynamic operation. The lowest current curve (yellow) was obtained in buffer, followed by the response obtained in YP medium and at different glucose concentrations starting from 1 mM, over 5 mM to 10 mM. The steps between the indicated concentration values of 10 mM, 50 mM, 100 mM and 150 mM are 10 mM. **(C)** Average standard deviation in % per data point of glucose concentrations measured with the biosensor platform (internal duplicate) in the three media under investigation. **(D)** Average time until signal stabilization of biosensor measurements in min in the three media under investigation.

The calibration curves obtained from glucose measurements in the three different media (buffer, YP medium, and YP medium containing 9 g/l NaCl), applying dynamic and static operation, are shown in Figure 2A. Additionally, the raw signal development under dynamic operation, exemplarily shown for glucose concentration measured in YP medium (the fermentation medium), the average standard deviation per measurement and the average time until signal stabilization in three different media are shown in Figures 2B–D.

Note that, in Figure 2A, under static operation glucose concentrations in YP medium (both with and without additional salt) were only investigated up to a glucose concentration of 50 mM and 80 mM, respectively. Aiming at on-line glucose monitoring, the operation without flow was a matter of curiosity and not in particular relevant to the final application. Hence, when the steady-state current seemed to reach a plateau during the experiment, the measurements of further samples were neglected. However, full calibration profiles from 1 mM – 150 mM glucose in all three media were investigated applying flow operation, as this was considered in particular relevant for the on-line application. The inspection of Figure 2 leads to three findings:

1. As can be seen in Figure 2A, the biosensor response for different glucose concentrations was very similar in the recommended buffer solution and YP medium, independent of the measurement being performed with or without flow. The addition of NaCl to the YP medium significantly lowered the overall signal profile and reduced twice the current value corresponding to 150 mM glucose concentration compared to the current value obtained in YP medium and buffer samples. This decrease in sensitivity might be explained by a chloride monolayer adsorbed onto the electrode surface, hindering the electrochemical kinetics. Monolayer coverage of chloride ions on platinum electrodes can already occure at chloride concentrations of 100 mM (Patil et al., 2011). Since in the present work, around 150 mM chloride ions were present and halide ions generally show a strong tendency to adsorb on platinum, it is likely that monolayer adsorption of chloride ions on the platinum electrode increased the resistance of the system, thus reducing the biosensor sensitivity. However, it is well-known, too, that an excessive amount of chloride ions is crucial to the operation of the reference Ag/AgCl electrode (Sophocleous and Atkinson, 2017).
2. Independent of the operation mode, the overall trend in each medium stayed the same, i.e., the current increased with increasing glucose concentration but showed different slopes or sensitivities, respectively, in the different media (Figure 2A). However, under static operation, the standard deviation per measurement and the time until signal stabilization were clearly dependent on the medium in which the measurement was performed (Figures 2C,D). Under static operation, the lowest st. dev. was obtained for glucose measurements in buffer solutions (2%), followed by a significant increase when the measurement was performed in YP medium (3.1%) and in YP medium containing additional salt (3.3%). The same trend applied to the average time until signal stabilization, which was lowest in buffer (3.1 min) and increased to 5.6 min and 7.0 min, respectively, when glucose was measured in YP medium and YP medium containing additional salt.
3. As expected, the application of flow during the measurement was favorable over static operation. In all three media, the average st. dev. per measurement and the average time until signal stabilization were significantly improved when applying flow (to less than 1% and 2.5 min, respectively) and showed a clearly reduced dependency on the medium itself (Figures 2C,D). The enhanced performance of the biosensing platform under flow operation can be explained by an increased mass transfer of glucose to the enzymatic layer due to the reduction of the diffusion layer on top of the hydrogel membrane. Besides, additional protons produced during peroxide decomposition are flushed out, thereby avoiding a local acidification that potentially can decrease the activity of the enzyme.

**FIGURE 3:**
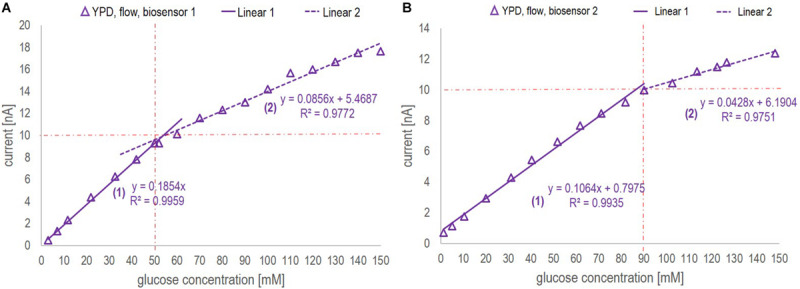
Calibration curves ranging from 1 to 150 mM glucose concentration obtained in YP medium applying a flow rate of 0.2 ml/min for *biosensor chip 1* **(A)** and *biosensor chip 2* **(B)**. The red dotted lines indicate the division points between the low (1) and high (2) glucose concentration range. The decision, which section of the curve to apply was based on the current value. For both calibration curves, the critical current corresponds to 10 nA.

The sensor is rated by the company for glucose quantification in solutions with a glucose concentration ranging between 0.05 and 60 mM. This also appeared to be the reliable linear range in which the current – glucose concentration correlation was found to be nearly independent of the medium used and the mode of operation (flow vs. no-flow). However, when applying a segmented calibration curve for low and high glucose concentrations, as presented in Figure 3, the measurement range could be remarkably increased up to glucose concentrations of 150 mM.

Since this study was aiming at on-line application of the biosensor platform during a fermentation process performed in YP medium, the calibration curves were based on the signal profile obtained in YP medium applying flow. The calibration curves of biosensor chip 1 and 2 are presented in Figure 3. By segmentation of the calibration curve into two sections approximated by linear regression, the full glucose concentration range typically found in various fermentation processes (0 – 150 mM) could be covered without the necessity of sample dilution. For both biosensor chips, the *R*^2^ value for the low and high range calibration curve was greater than 0.97, hence suggesting that glucose quantification could be performed reliably on-line. Further investigation comparing the calibration curves obtained with biosensor 1 and 2 is discussed with regards to batch-to-batch variability as part of section “Biosensor Chip Stability and Batch-to-Batch Variability.”

### At-Line Application of the Biosensor Platform During a Fermentation Process

Biosensor chip 1, previously calibrated in YP medium applying flow (Figure 3B), was applied at-line yielding operational real-time data for glucose measurements of distinct fermentation samples hourly.

**FIGURE 4:**
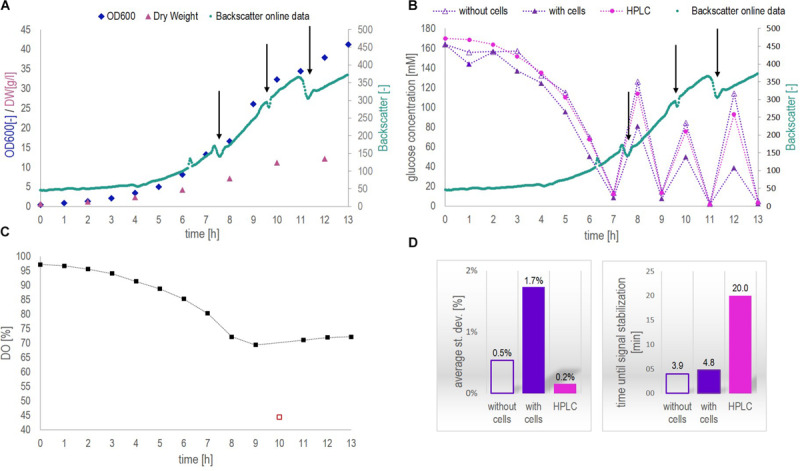
Data collected from *fermentation 1* during at-line application of biosensor chip 1. **(A)** Biomass concentration as a function of time measured by optical density (OD600, at-line) and dry weight (DW, off-line) as well as continuously on-line via the CGQBIOR (backscatter units). **(B)** Glucose concentration measured at-line with and without cells by means of the biosensor platform as well as off-line (cell-free samples) by HPLC. The addition of glucose during the fermentation is indicated by black arrows. Besides, the increase in biomass over time is demonstrated by backscatter measurements as in Figure 4A. **(C)** Dissolved oxygen (DO) profile over the fermentation course. The red square must be considered as an outlier. **(D)** Average st. dev. and average time until signal stabilization (time until measurement result) of the different glucose measurements performed.

Figures 4A–D show the results obtained from *fermentation 1* (Table 1). The progress of the fermentation is presented by the increase of the cell concentration and the decrease of glucose concentration over a 13 h period (Figures 4A,B). Besides, the level of dissolved oxygen (DO) was monitored continuously during the entire fermentation process (Figure 4C). The glucose concentration was followed by means of the biosensor platform measuring both, cell-free and cell-containing samples and the glucose profiles obtained were compared to HPLC results. Note that, only cell-free samples can be analyzed by HPLC as cell-containing samples would immediately block the capillaries of the system and the column used for separation. Figure 4D compares the performance of the biosensor platform with HPLC analysis regarding the average st. dev. and the average time until signal stabilization (average time until measurement result, respectively, regarding HPLC measurements).

During this fermentation, samples contained up to 12 g/l dry weight (Figure 4A). Backscatter data yielded a continuous trajectory of the growing biomass over the entire fermentation course, besides indicating the addition of glucose (signal drop due to local dilution of the fermenter content during spiking, Figures 4A,B). The signal drop in the DO profile at 10 h (red square) in Figure 4C must be attributed to a disturbance in the air supply line. The results presented in Figure 4B clearly indicated that glucose concentrations in the cell-free supernatant could be measured accurately by means of the biosensor platform during the entire fermentation process and were in agreement with HPLC results. Glucose measurements above 20 mM glucose concentration in cell-containing samples resulted in generally lower values compared to the concentration obtained from cell-free samples. For glucose concentrations above 20 mM and cell concentrations below 5 g/l, the off-set between cell-free and cell-containing samples amounted to minus 10%. This off-set increased to about minus 40% when a dry weight concentration of 5 g/l was exceeded (after 7 h). However, for glucose concentrations below 20 mM measured in cell-containing samples, no such an off-set was observed. As can be seen in Supplementary Figure S3, cell-containing samples analyzed with the biosensor platform after 4, 5, 6, 8, and 12 h clearly showed a current decrease at a certain time of measurement and did not reach steady state. Contrarily, the signal developed fully and reached steady state when measuring cell-free samples (Supplementary Figure S4). Bearing in mind that, when measuring cell-containing samples, both the cells and the biosensor consume oxygen, therefore, it seems likely that at glucose concentrations above 20 mM and cell concentrations above 5 g/l, the oxygen was consumed before the measurement with the biosensor platform could reach steady state. This was confirmed by studying the oxygen consumption rates in cell-containing fermentation samples (see section “Continuous On-Line Glucose Monitoring by the Biosensor Platform During a 10 h Yeast Fed-Batch Fermentation”). Moreover, it is important to observe that the DO inside the fermentation broth decreased from 100 to 70% (Figure 4C) during the process due to the increased cell concentrations and thus increased respiratory activity, hence decreasing the oxygen availability for the biosensor measurement. Electrochemical GOx-based biosensors require a minimum oxygen availability during operation to yield accurate measurement results. A single-use biosensor previously described in literature required at least 10 μM of dissolved oxygen for accurate quantification of 40 mM glucose in cell-free samples, (Moser and Jobst, 2013). Generally, oxygen limitation hampers the signal development within the biosensor and thus, the current signal cannot reach the steady state condition. This was the case for glucose concentrations above 20 mM and cell concentrations above 5 g/l dry weight. For glucose concentrations below 20 mM, no limitations attributed to oxygen availability (minimum DO level of 70% inside the fermenter) could be observed within this fermentation (reaching a maximum DW concentration of 12 g/l).

According to Figure 4D, both the average st. dev. and the time until signal stabilization increased for cell-containing samples compared to cell-free samples, from 0.5 to 1.7% and, respectively, from 4 to 5 min. This increase for both parameters could be expected. The presence of cells did not only add a solid phase to the system thus increasing its complexity and limiting the diffusion of glucose through the membrane toward the enzymatic layer, but also created the competition for glucose and DO inside the biosensor chip. In other words, due to the oxygen consumption by the cells, the oxygen transfer rate inside the biosensor chip was decreased, which slowed down the response time of the biosensor. However, all glucose measurements performed with the biosensor platform were subject to an average st. dev. of less than 2% and results were obtained in less than 5 min. On average, HPLC measurements showed a 10 – fold decreased st. dev. of 0.2% but a 4-fold increased time per measurement to obtain a result (considering only the HPLC protocol as such and no sample preparation time including sample filtration, acidification and filling the sample into HPLC vials).

A notable amount of (by) products such as ethanol and various other alcohols, glycerol, acetic acid, aldehydes and carboxylic acids are produced during the fermentation process, as demonstrated by the GC-MS in Supplementary Figure S5. Importantly, the glucose measurement by means of the biosensor platform was independent of the presence of various different electroactive species and changes in the medium emphasizing the advantage of operating a sensor with a highly selective and specific bioreceptor (enzyme, GOx).

### Continuous On-Line Glucose Monitoring by the Biosensor Platform During a 10 h Yeast Fed-Batch Fermentation

Biosensor chip 2, previously calibrated in YP medium applying flow (Figure 3B), was utilized on-line yielding continuous glucose monitoring during *fermentation 2* (Table 1).

**FIGURE 5:**
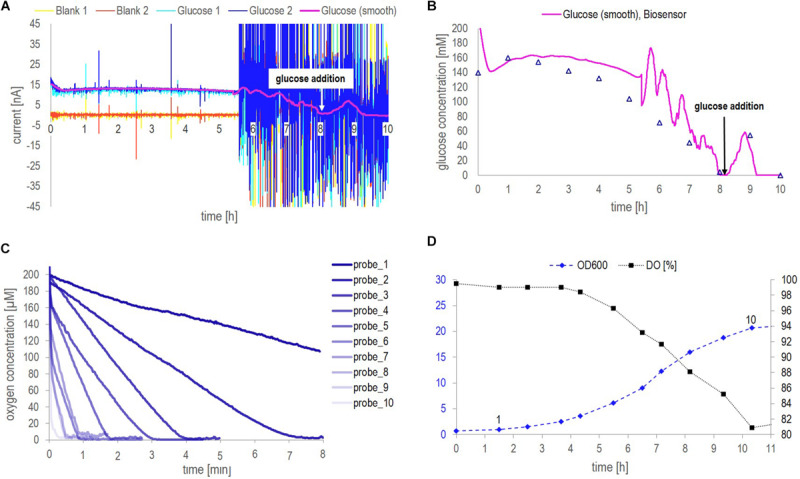
Data collected from *fermentation 2* during on-line application of biosensor chip 2. **(A)** Continuous raw signal of the biosensing platform, current in nA as a function of the fermentation time in min, as well as the smoothed raw current signal obtained from a Matlab^®^ in-house smoothing function. **(B)** Comparison of the glucose concentrations measured with the biosensor platform (based on the smoothed raw current signal and the calibration curve obtained in YP applying flow (Figure 3B) and HPLC analysis of distinct fermentation samples. **(C)** Dissolved oxygen concentration over time measured with the optical minisensor off-line for manually withdrawn, cell-containing fermentation samples. The probes are numbered from 1 to 10 corresponding to their acquisition time as indicated in the OD600 profile [panel **(D)**]. **(D)** Yeast growth indicated as OD600 together with the DO as a function of the fermentation time in hours. The numbers above the OD600 data points indicate the time point of probes 1–10 in panel **(C)**.

The data collected during *fermentation 2* applying the biosensor platform on-line is presented in Figure 5. It included the raw signal of the biosensor, the glucose signal of the biosensor, as well as OD600 and DO profiles for the fermentation under study (Figures 5A,B,D). Additionally, the oxygen consumption in cell-containing samples taken manually after distinct time points is shown in Figure 5C. This was done in order to prove the hypothesis stated in section “At-Line Application of the Biosensor Platform During a Fermentation Process,” that the high off-set observed between at-line measurements performed with the biosensor platform at high cell (>5 g/l) and high glucose concentrations (>20 mM) compared to HPLC results could be explained by oxygen limitations occurring due to competitive consumption of oxygen by both, the cells and the biosensor.

The raw signal obtained from glucose measurements performed with the biosensor platform became especially noisy after approximately 5.5 h. It is assumed that this noise as well as current spikes observed in the signal before 5.5 h can be attributed to an air bubble stuck in the biosensor chip, or, respectively, air bubbles passing through the biosensor chip. As no precaution regarding the sampling of air (inherently occurring in aerated fermentation broth) was taken, keeping the setup simple, small air bubbles could be observed in the tubing of the circulation loop and caused the noise observed. Small air bubbles passing the system seemed to be a minor disturbance (until 5.5 h) whereas an air bubble stuck inside the electrochemical cell (after 5.5 h) can evidently cause tremendous noise making it difficult to observe the signal trend. However, the actual trend of the glucose signal could be recovered by applying an in-house Matlab (version R2016a) smoothing function using differential filtering as described by Eilers (2005). Besides, variation in the smoothed glucose signal except for the peak resulting from glucose addition (after approx. 8 h) must be considered as artifacts resulting from the tremendous noise which could not be filtered by the smoothing function. If no mathematical filtering (smoothing functions) can be applied to the raw signal, the sampling of air can easily be avoided by adding e.g., a 20 μm stainless steel filter cap to the sampling port. Generally, the glucose signal trend was captured accurately and was in good agreement with HPLC results (Figure 5B). Interestingly, the glucose signal measured by the biosensor platform was constantly a bit higher compared to HPLC analysis. This might be due to the noisy raw signal as such, however, a lower sensor signal compared to HPLC measurements would rather be expected due to the presence of cells as described with respect to Figure 4B.

As described with respect to Figure 4B, cell-containing samples analyzed at-line did run into oxygen limitations when a glucose concentration of 20 mM and a cell concentration of 5 g/l DW were exceeded. A cell concentration of 5 g/l DW corresponded to an OD600 value of approx. 7 (Figure 4A). During this fermentation an OD600 value of 7 was reached after approximately 6 h (Figure 5D). The DO concentration in the corresponding probe 6 was 95 μM in the beginning of the measurement and the DO was consumed in less than 1 min (Figure 5C). Contrarily, the signal stabilization time of the respective (cell-free) samples was around 4 min (Figure 3D and Supplementary Figure S3). Note that, the signal stabilization time of cell-containing samples is expected to be even longer than 4 min, according to the observation in Figure 4D. Hence, the obtained data showed that the signal stabilization time of corresponding at-line measurements was higher than the period in which oxygen was present in cell-containing samples. In other words, cells consumed the oxygen before the measurement was finished. This confirmed that the measurement with the biosensor platform did run into oxygen limitations, restricting the signal development, due to concurrent consumption of oxygen by the cells and the GOx-layer inside the biosensor chip, especially when higher glucose and cell concentrations were present. The dissolved oxygen is depleted when measuring at-line samples, as these samples are no longer actively aerated. However, inside the fermenter, the dissolved oxygen concentration was maintained above 70% of saturation (Figures 4C, 5D), due to a constant air supply. Thus, oxygen limitations were not experienced during the on-line measurements with the glucose biosensor.

### Biosensor Chip Stability and Batch-to-Batch Variability

**FIGURE 6:**
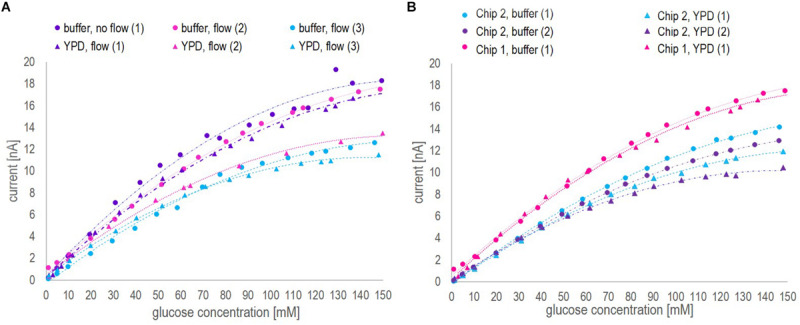
Different calibration profiles ranging from 1 to 150 mM glucose concentration obtained with biosensor chip 1 and 2, presenting the current [nA] as a function of the glucose concentration measured by HPLC [mM]. **(A)** Calibration profiles obtained with the electrochemical platform using biosensor chip 1 in buffer and YP medium before (1) and after (2) at-line application on fermentation samples, and after a 3 months storage period (3). **(B)** Calibration profiles obtained with the electrochemical platform using biosensor chip 2 in buffer and YP medium before (1) and after (2) on-line application during the fermentation. Moreover, the results of biosensor chip 1 and 2 are compared regarding the initial calibration in buffer and YPD medium.

In order to study the biosensor chip stability and batch-to-batch variability, biosensor chip 1 and 2 were re-calibrated after various measurements of fermentation samples (performed at-line or on-line, respectively). Besides, chip 1 was re-calibrated again after a 3 months storage period. Figure 6 shows the calibration profiles obtained as a function of usage and time with both biosensors. Besides, to conclude the calibration results obtained with chip 1 and 2, Figure 3 was investigated in detail, supplementing the results shown in Figure 6.

Inspection of Figure 3 and Figure 6 led to three conclusions.

1. The correlation between the current and the glucose concentration is similar for the two biosensors in buffer and YP medium but definitely sensor (and medium) dependent. The first biosensor showed an around 75% higher sensitivity (0.1854 nA/mM glucose, Figure 3A) than the second one (0.1064 nA/mM glucose, Figure 3B) with respect to the low range section of the calibration curve (Linear 1 in Figure 3). Besides, the division between low and high range calibration curve was 50 mM for the 1^st^ and 90 mM for the 2^nd^ biosensor. However, in both cases the shift in slope was observed at a critical current of 10 nA. The *R*^2^ value of the low and high range calibration curve of both biosensors was with 0.99 and 0.97, respectively, of a reliable accuracy. Generally, the sensitivity toward glucose was found to be higher for a concentration up to 60 mM (the recommended upper analyte detection limit by the company), yet, remarkably, the biosensors could be applied reliably for glucose concentrations up to 150 mM. The batch-to-batch variability with respect to calibration can mainly be linked to different enzyme activities and/or quantities immobilized on the electrodes. To validate the critical current for calibration curve division, the measuring range may be classified by the use of a biosensor on-line analyzer for bioprocess control as e.g., the BioPAT^®^ Trace Glucose/Lactate Analyzer (covering a glucose concentration range from 0 to 40 g/l).
2. The sensitivity for glucose concentrations measured in buffer, especially for glucose concentrations in the higher range, was consistently slightly higher compared to glucose concentrations measured in YP medium (Figure 6). This might be explained by a change of the diffusive properties of the membrane being decreased when large molecules like peptides are present as was the case in the complex YP medium. Large molecules might close pore structures and/or attach to the surface thus increasing the diffusive barrier to the electrode.
3. The sensitivity decreased as a function of usage and time (Figure 6). With respect to the biosensor’s application on fermentation samples, even after 10 h continuous use, the sensor was active and could be reused as represented in the respective calibration curves performed before and after the fermentation samples. The loss of sensitivity can be explained by a loss of enzyme activity or, supposedly, changes in the membrane characteristics. The operational stability stated by the company at 37°C is >2 weeks in continuous operation when measuring glucose concentrations up to 25 mM.

Finally, since the design of the used biosensor chip is patented and hence not conversant (it may be a bulk metal electrode, sputtered film or nanoparticulated surface), its behavior in terms of mechanical stability in the presence of fermentation samples was investigated by ICP-MS.

Previous studies on the operation of biosensors revealed different behaviors (mechanical stabilities) under operation. The leakage of iron ions from the Prussian Blue layer of 1^st^ generation biosensors was observed by Semenova et al. (2018, 2019). The authors investigated the mechanical stability of the hydrogen peroxide-specific catalyst, namely Prussian Blue, and measured the leakage in terms of iron irons by means of ICP –MS. It was shown that already the loss of iron ions at the ppm level of concentration of the hydrogen specific catalyst strongly affected the biosensor response and overall stability of the system. Moreover, the stability of such catalyst, which is the Pt-based working electrode in the biosensor under study, plays a crucial role in the fast hydrogen peroxide degradation, which prevents the deactivation of the enzyme and ensures the robustness of the glucose biosensor measurements. Contrarily, for one-step designed palladium-nanoparticles (Pd-NPs) assisted nanobiosensors, almost no leakage of Pd-NPs was detected (Semenova et al., 2020).

Therefore, in order to monitor the possible leakage of metal ion species from the biosensor platform under study (the Pt working electrode functions as a H_2_O_2_ specific catalyst itself, and hence its leakage would substantially decrease the biosensor’s activity), several glucose samples were taken during re-calibration of biosensor chip 1 and 2 and tested by means of ICP-MS (Table 2).

**TABLE 2:** Content of free Pt and Ag species found in tested glucose solutions during operation of the biosensor platform (Table 1, biosensor chip 1 and 2, point 3) as a result of ion migration from the electrode material detected by ICP-MS.

**Calibration**	**Pt_195_, ppb (st. dev., %)**	**Ag_107_, ppb (st. dev., %)**
Biosensor chip B.LV5 based on Pt and Ag as electrode materials	*y* = 40.47⋅*x* + 662.37 *R*^2^ = 0.9998 0 ppt (−0.946 ppt) (10.02)	*y* = 56.27⋅*x* + 3556.48 *R*^2^ = 0.9999 0 ppt (−60.03) (2.13)

*Negative concentration values obtained by use of the respective calibration curves were set to zero.*

Notably, no leakage of metal ions, i.e., Pt or Ag was observed for the biosensor operated at the used experimental conditions (Table 1, biosensor chip 1 and 2, point 3). This fact can be strongly considered as a proof for both the excellent mechanical and operational stability of the adapted biosensing platform.

## Discussion

Within this study, a commercial biosensor platform for glucose detection designed as a flow-through-cell was tested with respect to glucose detection in yeast fermentation samples. The novel application toward fermentation processes yielded accurate results and the platform showed outstanding stability properties. The fast and accurate measurement of glucose concentrations in fermentation samples obtained by the biosensor platform clearly supported its adaption as a time and resource minimizing alternative compared to HPLC analysis. The biosensor platform is considered as an accurate, robust, simple and inexpensive tool for glucose monitoring that can be readily applied in fermentation processes. Remarkably, the biosensor platform covered a detection range for glucose concentrations up to 150 mM, when applying a segmented calibration curve for low and high glucose concentrations. The calibration results suggested a current of 10 nA as a division point marking the switch between low and high glucose detection range. Besides, the biosensor platform showed outstanding mechanical stability under operation and provided accurate glucose quantification in the presence of various electroactive species inside the fermentation medium. In other words, present electroactive species did not interfere with the glucose measurement, which is otherwise an often outspoken concern with respect to the application of 1^st^ generation glucose biosensors toward complex samples. The results presented were obtained with a non-sterile biosensor chip. However, no contamination was observed during the fermentation as confirmed by microscopy of a mid- and end-fermentation sample. It is important to note that a sterile version of the biosensor is commercially available. Sterility is a major requirement in most bioprocesses, and further studies dedicated to this matter applying the sterile version of the biosensor chip will be helpful to analyze this important aspect of biosensor operation in more detail.

As learned throughout this work, calibration has to be performed in a representative fermentation matrix as glucose measurements in different media were found to be matrix dependent. Moreover, air bubbles in the biosensor flow-through-cell and oxygen limitations during operation of the biosensor platform need to be avoided. Within this study, at-line measurements of cell-containing samples were subject to oxygen limitation when high glucose (>20 mM) and high cell concentrations (>5 g/l DW) were present. However, this might have been avoided if a higher flow rate (>0.2 ml/min) was applied during at-line operation, increasing the mass transfer of glucose to the enzyme layer and thus reducing the time until signal stabilization. Besides, the combination of high glucose and high cell concentrations must be considered as an exception in the frame of fermentation processes. In this study, they were induced intentionally by glucose spiking during the fermentation process to challenge the biosensor platform particularly with respect to high glucose and cell concentrations simultaneously. Usually, high glucose concentrations are found in the beginning of batch fermentation processes when the cell concentration is low. For economical and metabolic reasons, feeding strategies are ideally designed to keep the glucose concentration in the broth as low as possible, feeding only as much glucose as is instantly consumed by the microorganisms. To avoid oxygen limitations on-line, a very close position of the biosensor platform to the sampling outlet of the fermenter is favorable, thus constantly supplying fresh, aerated broth during the measurement. Air bubbles disturbed the on-line application within this work. However, air bubbles can be easily avoided e.g., by applying a 20 μm filter cap to the sampling port.

The biosensor platform under study, commercially available and ready to use, facilitated the monitoring of the crucial parameter glucose practically with minimum effort. It evidently demonstrates that technology for the monitoring of crucial fermentation parameters is available, and already tremendously reduces the labor intensity in the laboratory. However, trust and knowledge on available technology can only be gained and deepened when considered on a daily basis. Thus far, we see the operation of the biosensor platform limited by the fulfillment of the minimum oxygen requirement during operation. Hence, it can only be applied to well-aerated (aerobic) fermentation processes, or, respectively, aerated samples. The oxygen availability inside the hydrogel membrane boils down to a sufficient mass transfer, which in turn, is limited by the low flow rate range applicable to microfluidic devices (0.1 – 1 ml/min for the sensor system under study). More application studies must be conducted to investigate the performance of the biosensor platform at e.g., high cell densities (rather low cell concentrations of maximal ca. 12 g/l dry weight were present within this study), which are expected to decrease the mass transfer further. Nevertheless, it is worth mentioning, that oxygen limitations were only observed at glucose concentrations above 20 mM, and feeding strategies are commonly designed to control the glucose level at very low concentrations inside the fermenter. Thus, high cell densities may not be an issue in such case. Besides, the biosensor’s activity over a period of several days of continuous operation will be interesting to study, which will answer concerns about the enzyme activity, or, respectively, the performance of the platform under continuous long-term application.

The confident use of applied research solutions starts in every laboratory before it will gradually find entry to industrial application and processing. Even more advanced tools might be available in the future. Nonetheless, convention and standards need to change gradually, otherwise a given opportunity might be lost on the way. By committing to the present technical progress, we can only benefit in terms of both, knowledge and understanding of the process and the instrumentation as such, pushing toward a generic monitoring tool. With this study we want to encourage both academic and industrial societies from relevant areas to commit to the current technical progress and to gain confidence in using commercially available but still rather uncommonly used biosensor technology as a cheap, ‘plug and play’ monitoring tool in fermentation processes.

## Data Availability Statement

The datasets generated for this study are available on request to the corresponding author.

## Author Contributions

KP conceived, designed and conducted the experimental research, analyzed the experimental data including the fermentation processes, the application of the biosensor platform, and reference HPLC analysis. DS conducted the analysis of oxygen consumption in cell-containing fermentation samples by means of the oxygen mini sensor and supported the choice of experiments performed. YS developed and applied GC-MS and ICP-MS assays to evaluate the matrix effects raised from YPD medium and to estimate the mechanical stability of the adapted sensing platform. KP wrote the manuscript. All authors contributed to scientific discussions, reviewed, and approved the final version.

## Conflict of Interest

The authors declare that the research was conducted in the absence of any commercial or financial relationships that could be construed as a potential conflict of interest.

## Footnotes

1 Conventionally, the calibration of an electrochemical biosensor is done by correlating the current at a defined time point (often at 90% conversion, not at steady-state) to the glucose concentration present. However, within this work, the stabilization time for each sample (respectively, the time of the measurement to reach steady-state) was dependent on the glucose concentration present, the cell concentration present (in fermentation samples) and the medium used (buffer or YP medium for calibration). Hence, the final current value was obtained by averaging the last 10 data points of the steady-state current. Within this work, this applies to every current value obtained from glucose measurements by the biosensor platform.

2 Describes measurements of samples withdrawn manually from the bioreactor performed off-line in parallel to the process. Data is obtained with a small time delay.

3 Describes measurements of samples withdrawn manually from the bioreactor, collected and stored until analysis. Data is obtained with some hours/days of delay.

4 Describes measurements performed with a sensor/probe yielding results in real-time practically with no time delay.
