# Supplementary material for: Automated Electrochemical Glucose Biosensor Platform as an Efficient Tool Toward On-Line Fermentation Monitoring: Novel Application Approaches and Insights
Source: Front Bioeng Biotechnol. 2020 May 21;8:436. doi: 10.3389/fbioe.2020.00436 (PMC7253623; doi:10.3389/fbioe.2020.00436)
Supplement: Supplementary file 1 [file Data_Sheet_1.PDF]

Supplementary Material

Figures

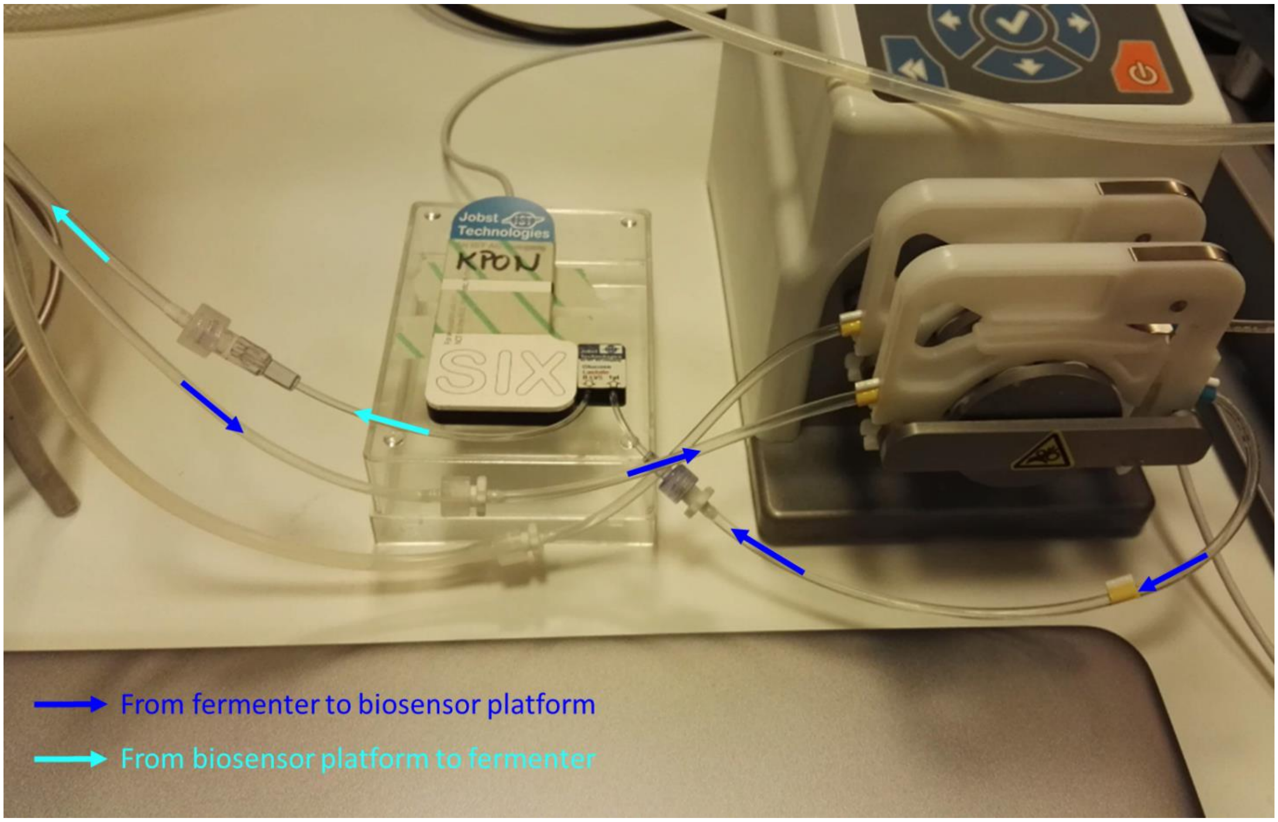

Figure S1*: Biosensor platform connected via a pump to the fermentation set-up.*

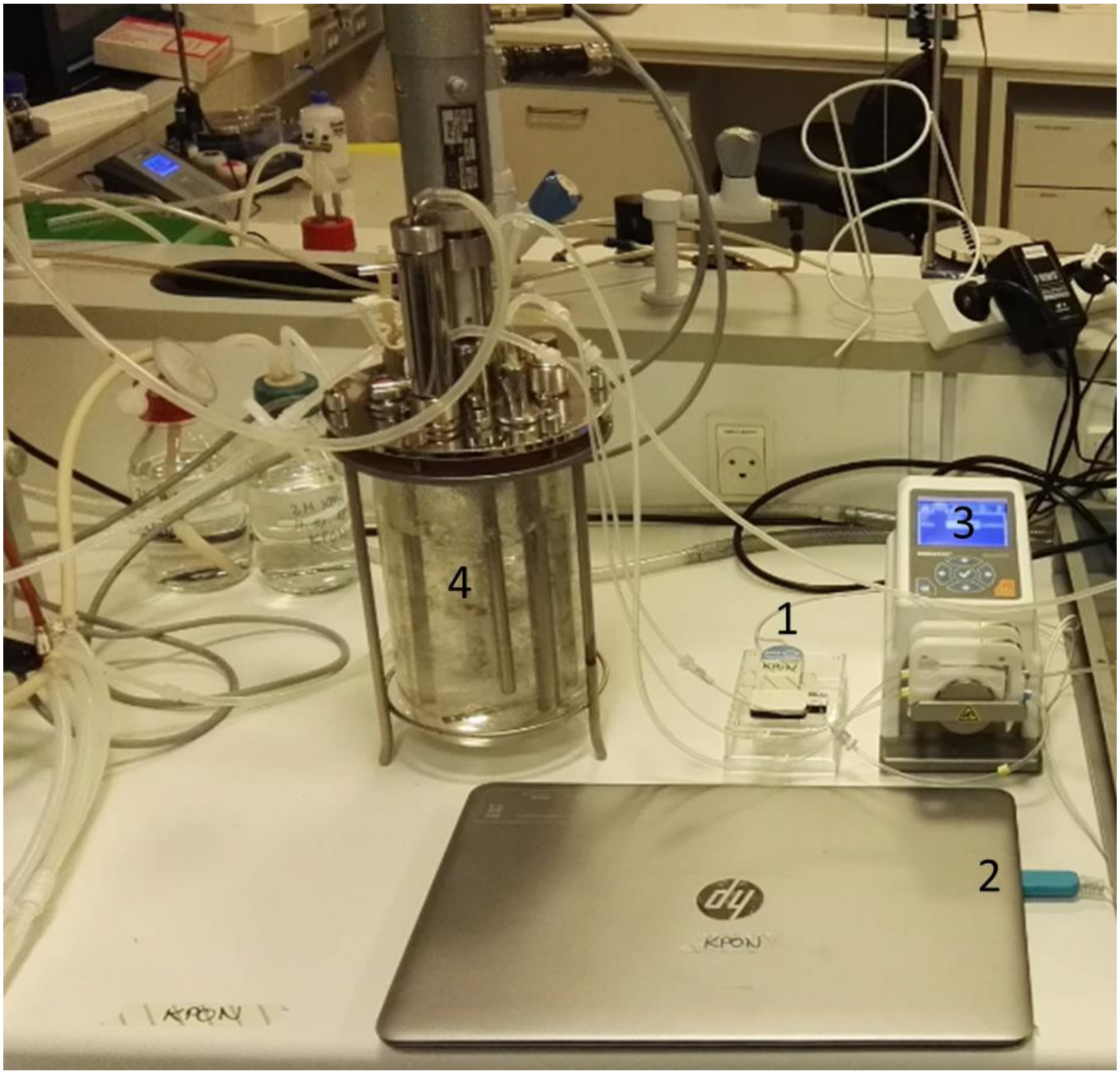

***Figure S2**: Biosensor platform connected to the fermentation set-up. 1: Biosensor platform. 2: Computer with connected USB cable coming from the Biosensor platform (SIX server). 3: Pump for recirculation of the fermentation broth. 4: Fermenter.*

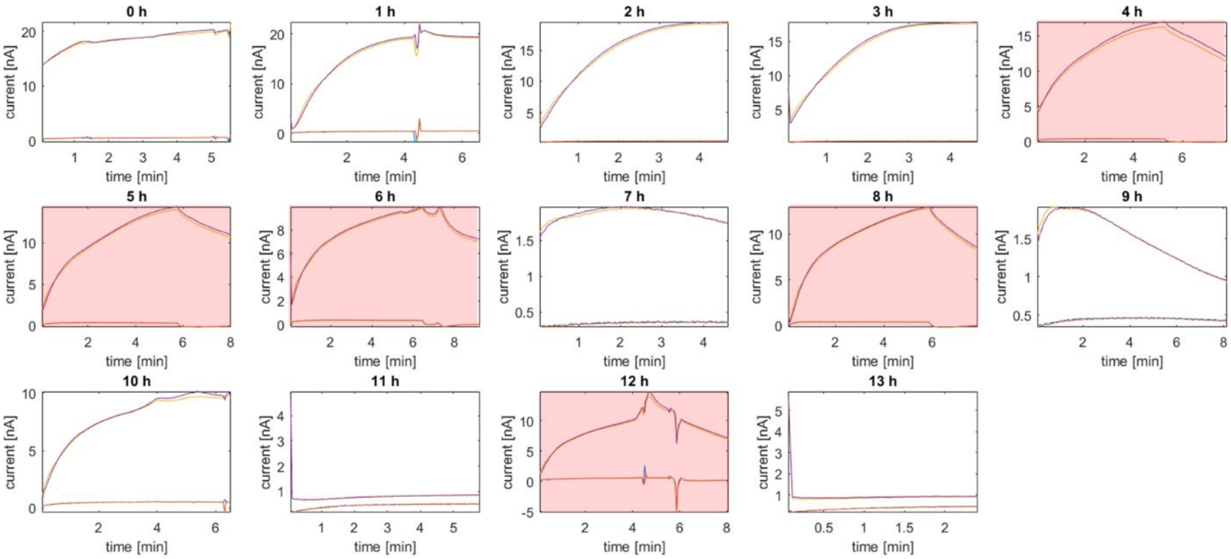

***Figure S3**: Raw signal development of <u>cell-containing fermentation samples</u> (relative sampling time indicated above each graph) measured by the biosensor platform, applying a flow rate of 0.2 ml/min. The measurements marked in red did not reach steady state and hence, the glucose concentration was obtained from the curve maximum and does not represent the actual glucose concentration.*

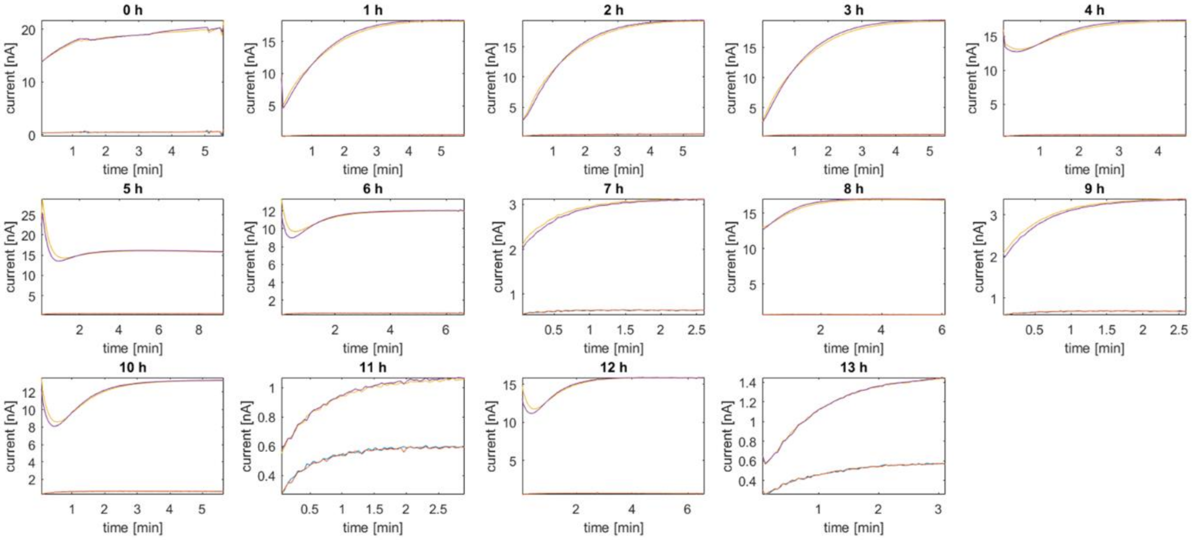

***Figure S4**: Raw signal development of <u>cell-free fermentation samples</u> (relative sampling time indicated above each graph) measured by the biosensor platform, applying a flow rate of 0.2 ml/min. Each measurement reached steady state, thus accounting for an accurate glucose measurement.*

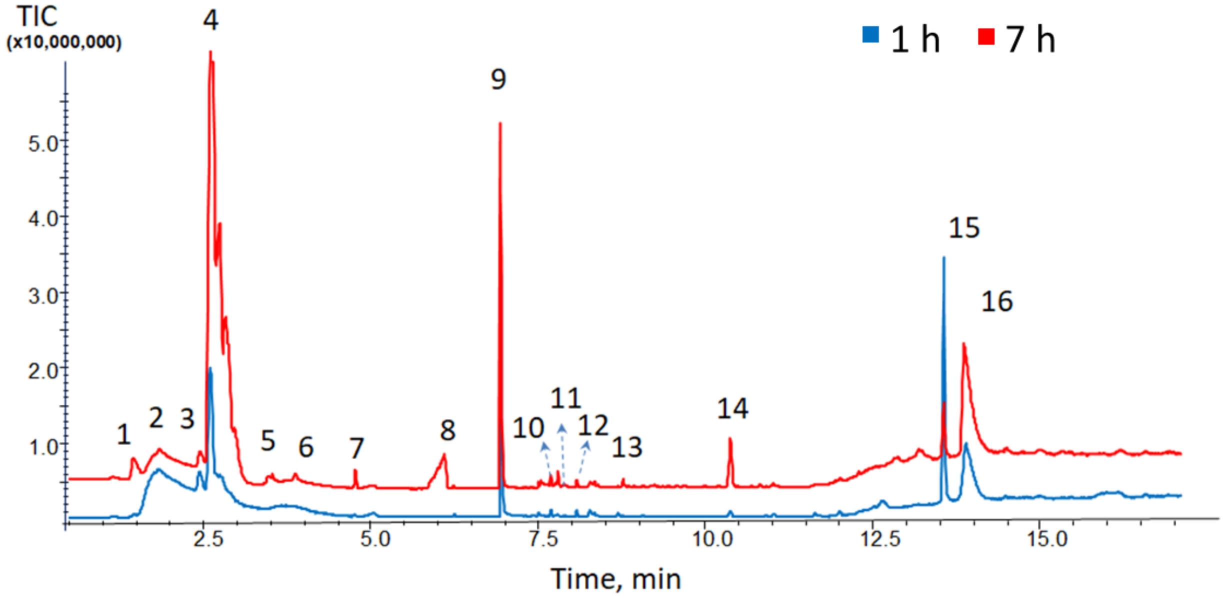

Figure S5: *GC-MS chromatograms obtained from the ZB-WAX plus column indicating the changes in the fermentation (YPD) medium over time as a result of the microbial activity, exemplarily demonstrated for samples taken after 1 and 7 h during fermentation 2. Peak assignments: 1 – carbon dioxide; 2 – acetaldehyde; 3 – butanal; 4 – ethanol; 5 – 2-butenal; 6 – isobutyl alcohol; 7 – 1-3methyl butanol; 8 – 3-hydroxy, 2 – butanone; 9 – acetic acid; 10 – propanoic acid; 11 – 2,3-butanediol; 12 – 1,3 butanediol; 13 – butanoic acid; 14 – phenyl alcohol; 15 – 2-formy-1-methylpyrrole; 16 – glycerol. Note: ethanol, acetic acid and glycerol are the typical main byproducts of a yeast fermentation produced from glucose and secreted into the medium due to overflow metabolism.*
